# Gut Dysbiosis and the Molecular Landscape of the Gut–Skin Axis: Comparative Insights and Therapeutic Implications for Atopic Dermatitis and Psoriasis

**DOI:** 10.3390/cells15070594

**Published:** 2026-03-26

**Authors:** Klara Andrzejczak, Emilia Kucharczyk, Agata Sternak, Tomasz Busłowicz, Małgorzata Ponikowska

**Affiliations:** 1 Faculty of Medicine, Wroclaw Medical University, Wybrzeze L. Pasteura 1, 50-367 Wroclaw, Poland; 2 University Centre of General Dermatology and Oncodermatology, Wroclaw Medical University, 50-556 Wroclaw, Poland

**Keywords:** gut–skin axis, atopic dermatitis, psoriasis, chronic inflammatory skin diseases, microbiota-derived metabolites, short-chain fatty acids (SCFAs), aryl hydrocarbon receptor (AhR), gut dysbiosis

## Abstract

Chronic inflammatory skin diseases, including atopic dermatitis (AD) and psoriasis, are systemic immune-mediated disorders driven by dysregulated immune responses. The gut–skin axis is a bidirectional network linking intestinal microorganisms, their metabolites, and host immunity. It connects microbiome composition and function with systemic inflammation and cutaneous pathology, shaping disease-specific mechanisms such as Th2/IL-4/IL-13-mediated barrier dysfunction in AD and Th17/IL-23/IL-17-driven hyperproliferation in psoriasis. Microbiota-derived metabolites, including short-chain fatty acids, tryptophan-derived aryl hydrocarbon receptor ligands, and bile acid-dependent FXR/TGR5 signaling, modulate immune homeostasis and epithelial integrity. Gut dysbiosis, impaired metabolite production, and barrier dysfunction disrupt regulatory networks, amplifying inflammation. Microbiota-targeted interventions, including probiotics, synbiotics, postbiotics, and precision nutrition, may serve as adjunctive therapies, although further well-controlled clinical studies are needed. Integrating multi-omics, metabolomics, and functional microbial profiling, alongside investigations of the gut mycobiome and virome, will be critical to identify predictive biomarkers and optimize therapeutic strategies. These concepts remain mechanistically compelling but largely theoretical, requiring validation in longitudinal and interventional studies.

## 1. Introduction

### 1.1. The Changing Landscape of Inflammatory Skin Diseases: A Systemic Focus

Immune-mediated inflammatory diseases (IMIDs) represent a heterogeneous group of chronic conditions with complex pathogenesis, characterized by dysregulated immune responses and systemic inflammation. Chronic inflammatory dermatoses, including atopic dermatitis (AD) and psoriasis, serve as model IMIDs in which the skin acts both as a target organ and an active immune regulator. These prevalent conditions significantly reduce quality of life and are associated with systemic comorbidities [1,2,3,4].

As the largest organ of the human body, the skin serves as a key immunological barrier, protecting the host from mechanical, microbial, chemical, and allergenic factors. In AD and psoriasis, immune dysregulation drives chronic activation of proinflammatory pathways, increasing chemokine and cytokine expression. Persistent inflammation promotes comorbidities and may increase the risk of complications. A detailed understanding of the links between local and systemic immune mechanisms is crucial for developing novel therapeutic strategies [1,5,6]. The pathogenesis of atopic dermatitis and psoriasis is driven by distinct, though partially overlapping, immunological pathways [7].

AD is a chronic inflammatory skin disease characterized by recurrent eczematous lesions and pruritus. Its pathogenesis involves genetic predisposition, epidermal barrier dysfunction, and immune-mediated inflammation. AD is associated with systemic comorbidities, including the atopic march (food allergies, asthma, allergic rhinitis) and mental health disorders [7,8,9,10,11,12].

Psoriasis is a chronic inflammatory skin disease characterized by well-circumscribed, erythematous, scaly plaques. Its pathogenesis involves a strong genetic predisposition, with HLA-C*06:02 as a major risk factor, especially in early-onset cases. Psoriasis is associated with systemic comorbidities, including cardiovascular disease, metabolic syndrome, psoriatic arthritis (PsA), and mental health disorders [7,13,14,15,16].

Despite advances, mechanisms sustaining chronic immune dysregulation remain incompletely understood. Emerging evidence suggests that gut microbiota dysbiosis may modulate systemic inflammation and cutaneous immune responses, highlighting the gut–skin axis as a promising target for novel therapeutic strategies [4,5,11,17,18,19].

### 1.2. The Gut–Skin Axis Concept

The gut microbiome shapes innate and adaptive immunity and regulates physiological processes, including metabolism, intestinal barrier homeostasis, inflammation, and hematopoiesis. Immune homeostasis relies on complex, bidirectional communication between the microbiome and host tissues via neural, endocrine, and hematogenous pathways. In genetically predisposed individuals, disruption of this network under environmental stress contributes to the pathogenesis of many immune-mediated diseases [5,20,21,22].

The gut–skin axis, part of the broader gut–organ axis, is a dynamic network linking gut microbes and cutaneous immunity. This interaction is driven by immune modulation, systemic inflammation, and changes in microbiome composition and function, affecting inflammatory mediators and T-cell activity in both the gut and skin [5,21,23,24].

This relationship involves gut barrier dysfunction, activation of proinflammatory pathways, and systemic effects of microbial metabolites. Dysbiosis is observed in many inflammatory skin diseases, including AD and psoriasis, suggesting a role in disease pathogenesis and clinical course [2,24,25,26].

While most evidence highlights gut-to-skin effects, skin barrier damage can also alter gut microbiota and disrupt intestinal immune homeostasis, emphasizing the bidirectional nature of this axis [27].

### 1.3. Gut Microbiota in Homeostasis and Dysbiosis

The gut microbiota is a dynamic community of bacteria, archaea, viruses (virome), fungi (mycobiome), and phages, comprising 10^13^–10^14^ commensal microorganisms that continuously interact with the host immune system. Its composition and function are shaped by environmental factors, diet, lifestyle, and genetic predisposition. A balanced microbiota maintains gut barrier integrity, modulates systemic immune responses through Th17/Treg balance, and influences immune cell differentiation in the gut and distant organs, including the skin. Microbial metabolites, such as short-chain fatty acids, secondary bile acids, and tryptophan derivatives, serve as key mediators of this gut–skin communication [5,23,24,25,28,29,30,31,32].

Disruption of this homeostasis, intestinal dysbiosis, defined as an imbalance in the composition and functional activity of the gut microbiota, characterized by loss of beneficial microbes, overgrowth of potentially pathogenic species, and reduced diversity, leads to increased epithelial permeability and facilitates translocation of microbial components (e.g., LPS, peptidoglycans) into systemic circulation. These signals, together with altered neurotransmission, promote chronic inflammation and disturb skin homeostasis. Dysregulated effector and regulatory T-cell activity amplifies proinflammatory cytokine production, further impairing barrier function and creating a vicious cycle of systemic inflammation, contributing to chronic inflammatory skin diseases such as AD and psoriasis [5,23,29,31,32,33].

### 1.4. Aim of the Review

The gut–skin axis is a complex bidirectional system in which microbiota-derived metabolites, immune signaling, and systemic inflammation modulate cutaneous pathology in chronic inflammatory skin diseases such as atopic dermatitis and psoriasis.

This review aims to:

(1) Outline key molecular and cellular pathways linking gut dysbiosis to skin pathology, focusing on microbiota-derived signaling molecules, including short-chain fatty acids acting as HDAC inhibitors, tryptophan-derived AhR ligands, bile acid-mediated FXR/TGR5 signaling, and Toll-like receptor pathways;
(2) Compare immunological axes and microbiota signatures in AD and psoriasis to identify disease-specific mechanisms;
(3) Evaluate the translational potential of microbiota-targeted interventions as adjunctive therapies to standard treatments.

From an evidence-based medicine perspective, gut–skin interventions should be considered primarily as adjunctive therapies. This review integrates current molecular evidence, showing how gut-derived signals modulate cutaneous immunity and providing a mechanistic rationale for microbiota-targeted strategies.

## 2. The Gut–Skin Axis: Cellular Education and Molecular Signaling

The immune system protects the body from pathogens while maintaining tolerance to self and harmless antigens, and immune homeostasis depends on a balance between activation and suppression that prevents chronic inflammation. The gut microbiome is an important contributor to this equilibrium, as it educates immune cells, modulates inflammatory responses, and preserves intestinal barrier integrity, thereby limiting systemic translocation of microbes and their metabolites. Through these microbiota-driven mechanisms, both innate and adaptive immunity are regulated not only in the gut but also in distant organs, including the skin [11,31,34,35]. Dysbiosis has been associated with chronic immune-mediated inflammatory skin diseases such as atopic dermatitis and psoriasis [5].

In this context, microbiota-derived signals play a central role in shaping immune responses along the gut–skin axis.

### 2.1. Cellular Education in the GALT: From Innate Sensing to Adaptive Immune Regulation

#### 2.1.1. Antigen Sensing and Dendritic Cell Education in the GALT

Gut-associated lymphoid tissue (GALT), a major component of mucosal immunity, plays an important role in host–microbiota interactions, and its development and function are strongly shaped by the gut microbiota [31,34,36,37,38]. It links local microbial sensing with systemic immunity and may influence distant tissues, including the skin, through antigen-presenting cells (APCs) and circulating adaptive immune cells [34,36].

Key cellular components include antigen-sampling M cells, T and B lymphocytes, and innate immune cells such as dendritic cells (DCs) and macrophages [34,36].

Dendritic cells (DCs) bridge innate and adaptive immunity by recognizing microbial signals through pattern recognition receptors (PRRs), including Toll-like receptors (TLRs) and NOD-like receptors (NLRs). In the intestine, these receptors on DCs, macrophages, and epithelial cells detect both commensal-associated (MAMPs) and pathogen-associated molecular patterns (PAMPs).

M cells and goblet cells capture microbial antigens and deliver them to dendritic cells, while some antigens are sampled directly by transepithelial dendritic cells. DCs present antigens via major histocompatibility complex class II (MHC II) molecules to naive T cells, providing costimulatory signals and cytokines that shape T-cell polarization and effector function [35,39,40,41].

A hallmark feature of intestinal dendritic cells is their ability to promote immune tolerance by inducing regulatory T cells (Treg) through mediators such as transforming growth factor β (TGF-β) and the vitamin A metabolite retinoic acid (RA) [35,37,38,40,42,43].

Dendritic cells orchestrate T-cell differentiation toward Th1, Th2, Th17, or Treg subsets, contributing to immune homeostasis and tolerance. While these immunological pathways are well established, their modulation by microbiota-derived signals represents a key mechanism linking GALT activity with systemic immune regulation. Antigen characteristics and the local microenvironment determine T-cell polarization and regulatory T-cell expansion [36,41,44,45].

The intestinal microbiota further shapes immune tolerance by engaging PRRs, promoting immune cell differentiation and antimicrobial peptide (AMP) production. A balanced microbiome supports GALT function and contributes to the homeostatic balance between regulatory and effector T cells, thereby controlling inflammation in both the intestine and distant organs, including the skin. Disruption of this balance may contribute to chronic inflammatory dermatoses such as atopic dermatitis and psoriasis [29,31,32,40].

Resident Langerhans cells (LCs) provide local immune surveillance in the epidermis, while gut-primed T cells enable microbiota-driven immune signals to influence distal tissues such as the skin [11].

#### 2.1.2. Modulation of the Th17/Treg Balance for Systemic Immune Homeostasis

CD4^+^ T cells are central to adaptive immunity and play a key role in immune-mediated diseases. Upon stimulation through the T-cell receptor (TCR) and cytokines, naive T cells differentiate into Th1, Th2, Th17, or Treg subsets, guided by specific transcription factors [29,31,46].

The gut orchestrates T-cell migration to distal tissues, while the gut microbiota regulates the Th17/Treg balance, maintaining immune homeostasis. Th17 cells promote proinflammatory responses and defense against pathogens, whereas Treg cells suppress excessive immune activity and support tolerance [29,31].

Th17 cytokines, including IL-17 and IL-22, contribute to barrier integrity and tissue regeneration. IL-22 stimulates keratinocyte and fibroblast proliferation and induces antimicrobial proteins and chemokines that recruit neutrophils, enhancing innate immunity and wound healing. However, excessive Th17 activation and overproduction of IL-17 and IL-22 drive immune-mediated diseases such as psoriasis and atopic dermatitis.

Treg cells maintain homeostasis through IL-10, TGF-β, and IL-35, and their differentiation and function are modulated by microbiota-derived metabolites [29,30,31,47,48].

The Th17/Treg balance is regulated by transcription factors RORγt (Th17) and Foxp3 (Treg) and by cytokine signaling, with IL-6 and IL-23 promoting Th17 differentiation, whereas TGF-β and retinoic acid (RA) favor Treg development. Ultimately, naive CD4^+^ T-cell fate reflects the balance between RORγt and Foxp3 activity, together with cytokine and microbiota-derived signals [30,31,49,50,51,52,53].

Dysregulation of the Th17/Treg axis in both the gut and skin represents an important mechanism in chronic inflammatory dermatoses, perpetuating local and systemic inflammation. Microbiota-derived signals help maintain this equilibrium, highlighting the gut–skin axis as an important regulator of immune homeostasis in chronic inflammatory dermatoses such as psoriasis and atopic dermatitis [2,29,31,32,54,55].

**Figure 1:**
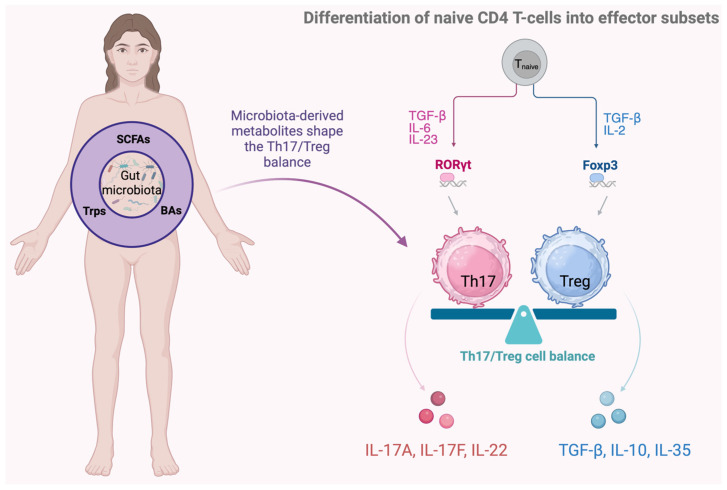
Impact of microbiota-derived metabolites (SCFAs, tryptophan derivatives and bile acids) on the Th17/Treg equilibrium and systemic immune homeostasis. Created in BioRender. Andrzejczak, K. (2026) https://BioRender.com/iavuza7.

**Figure 2:**
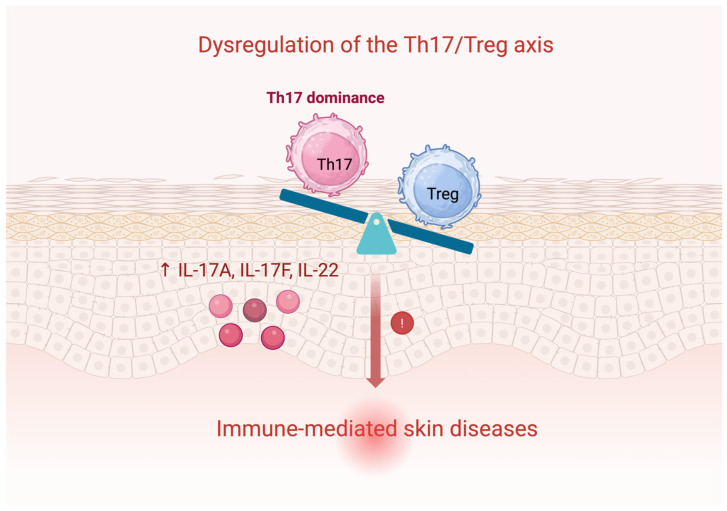
Dysregulation of the Th17/Treg axis and its importance in chronic inflammatory skin diseases. Created in BioRender. Andrzejczak, K. (2026) https://BioRender.com/7rsowo8.

Key interactions between gut microbiota-derived metabolites and the Th17/Treg balance are shown in Figure 1, while the Th17/Treg imbalance contributing to chronic inflammatory skin diseases is schematically presented in Figure 2.

#### 2.1.3. Immune Cell Trafficking and Skin Homing of CLA^+^ T Cells

Immune cell trafficking is essential for pathogen defense and immune tolerance, ensuring precise delivery of effector cells to specific tissues through a multistep adhesion cascade. T lymphocytes express homing receptors that recognize tissue-specific adhesion molecules and chemoattractants [56,57].

Circulating CLA^+^ (cutaneous lymphocyte antigen) T cells bind to E-selectin on endothelial cells, enabling skin homing. CLA is expressed on approximately 15% of peripheral T cells and contributes to local cutaneous immunity during inflammatory responses [56,57,58,59].

Retinoic acid (RA), a vitamin A metabolite produced by intestinal dendritic cells, stromal cells, and certain gut bacteria, modulates homing receptor expression. High RA levels in the GALT promote T-cell migration to the intestinal mucosa by upregulating α4β7 integrin and CCR9 while simultaneously inhibiting CLA expression and limiting skin homing [56].

Under certain conditions, activation by intestinal dendritic cells can generate “dual-tropic” CLA^+^α4β7^+^ lymphocytes capable of migrating to both intestine and skin. This process is enhanced by inflammatory cytokines such as IL-12 and IL-23, which promote skin-directed trafficking [56,60].

Following antigen encounter, some lymphocytes establish residence in peripheral tissues. In the skin, tissue-resident memory T cells (Trm) form a non-circulating population that remains at sites of prior antigen exposure, providing continuous immune surveillance and rapid protective responses. Trm complement CLA^+^ cells, contributing to long-term regulation of cutaneous immune responses [61,62,63].

Induction of intestinal immune tolerance may therefore mitigate skin inflammation by limiting uncontrolled migration of activated immune cells between gut and skin and reducing systemic dissemination of proinflammatory signals [56].

### 2.2. Microbiota-Derived Molecular Mediators and Cellular Sensing Along the Gut–Skin Axis

The gut microbiome is an important regulator of host immunity, inflammation, and metabolism and plays a significant role in the gut–skin axis. Alterations in its composition and function have been associated with increased inflammatory activity in the skin. Microbial metabolism generates numerous signaling molecules, including short-chain fatty acids (SCFAs), tryptophan derivatives (Trp), and bile acid metabolites (BAs), which enter the systemic circulation and modulate immune cell differentiation and function through receptor-mediated genetic and epigenetic mechanisms. Consequently, these metabolites act as major molecular mediators of the gut–skin axis, supporting intestinal barrier integrity and systemic immune homeostasis. In this context, gut dysbiosis may function as an immunometabolic amplifier, enhancing inflammatory signaling through microbiota-derived metabolites and immune pathways implicated in chronic inflammatory dermatoses such as atopic dermatitis and psoriasis [30,64,65].

#### 2.2.1. Short-Chain Fatty Acids (SCFAs): Epigenetic Regulation and Skin Barrier Integrity

SCFAs, primarily acetate (C2), propionate (C3), and butyrate (C4), are produced through microbial fermentation of dietary fiber and resistant starch in the cecum and colon. SCFAs exert immunomodulatory effects by regulating cytokine production in neutrophils, macrophages, dendritic cells, and T lymphocytes in a context-dependent manner [30,64,66,67,68,69].

SCFAs promote regulatory T-cell differentiation and the production of anti-inflammatory cytokines such as IL-10 and TGF-β, while also enhancing mitochondrial metabolism and strengthening epithelial barrier function through increased mucus and IgA secretion.

Butyrate, a natural HDAC inhibitor, increases histone acetylation at the *FOXP3* promoter, thereby inducing *FOXP3* expression and Treg differentiation. It also regulates Treg-inducing molecules in epithelial and dendritic cells and can stimulate retinoic acid production, which is essential for intestinal Treg generation. Propionate and acetate exert similar HDAC-inhibitory activity, enhancing lymphocyte function and IL-10 production [64,67,70,71,72].

The anti-inflammatory effects of butyrate include inhibition of NF-κB activation and stabilization of IκBα, thereby reducing the expression of proinflammatory cytokines such as IL-6 [73,74,75,76,77].

SCFAs act as ligands for surface and nuclear receptors, including GPR41/43, GPR109a, and PPARγ, thereby regulating immune responses and inflammatory processes [30,64,78,79].

Trompette et al. demonstrated that butyrate directly affects epidermal keratinocyte metabolism, accelerating their differentiation and enhancing skin barrier function. This increases resistance to allergen penetration, reduces sensitization, and lowers the risk of atopic diseases by stimulating the production of structural proteins and lipids in the stratum corneum. These effects depend on mitochondrial function and activation of the keratinocyte differentiation program [66].

#### 2.2.2. Tryptophan-Derived Indoles and AhR Signaling in Epidermal Barrier Integrity and Immune Modulation

Tryptophan (Trp) is an essential amino acid obtained from the diet and serves as a precursor to numerous biologically active metabolites. Gut microbiota metabolize Trp through three main pathways: the indole, kynurenine, and serotonin pathways.

The indole pathway produces bioactive derivatives such as indole and related compounds (e.g., indole-3-aldehyde, indole-3-acetic acid, and tryptamine), whereas the kynurenine pathway produces kynurenine (Kyn) and related metabolites. The serotonin pathway leads to the production of serotonin and its derivatives [30,80,81,82].

The aryl hydrocarbon receptor (AhR) functions as a sensor of microbiota-derived signals and plays a key role in maintaining host–microbiota homeostasis. It can be activated by various endogenous and exogenous ligands, including Trp-derived metabolites [30,80,83,84,85].

In the absence of ligands, AhR remains in the cytoplasm in an inactive complex with chaperone proteins. Upon ligand binding, the receptor undergoes conformational changes, translocates to the nucleus, and heterodimerizes with ARNT, enabling activation of target genes involved in immune regulation and barrier function [86,87,88].

AhR is highly expressed in mucosal tissues, where its activation enhances epithelial barrier integrity and modulates both local and systemic immune responses [70,83,89]. Indole metabolites also sustain the expression of functional IL-10 receptors in epithelial cells, which is essential for barrier homeostasis [81,82].

Additionally, Trp metabolites promote tolerogenic dendritic cells and Foxp3^+^ regulatory T-cell differentiation while modulating Th17/Th22 responses and macrophage polarization toward an anti-inflammatory phenotype [30,70,80,90,91]. AhR contributes to maintaining the balance between proinflammatory Th17 cells and immunosuppressive Tregs, underscoring its role in immune modulation [86]. Its activation also reduces proinflammatory IL-6 production, partly by inhibiting histamine release from macrophages [92].

In the skin, AhR is essential for homeostasis and epidermal barrier function. Controlled activation supports epidermal renewal, whereas excessive stimulation may induce oxidative stress and promote proinflammatory mediators. AhR also modulates cytokine signaling, including IL-10, IL-17, and IL-22, and regulates macrophage and dendritic cell function as well as lymphocyte survival. IL-17 and IL-22 contribute to chronic inflammatory skin diseases, whereas IL-10 exerts anti-inflammatory effects [86,88].

#### 2.2.3. Bile Acids: FXR/TGR5 Signaling and Immune–Metabolic Modulation

Bile acids (BAs) are synthesized from cholesterol in the liver via classical and alternative pathways. Primary BAs enter the intestine, where gut microbiota convert them into secondary forms such as lithocholic acid (LCA) and deoxycholic acid (DCA).

Beyond their role in lipid digestion, BAs act as signaling molecules with hormone-like effects. They regulate glucose and energy metabolism and modulate immune responses through nuclear and membrane receptors. Among these, the nuclear receptor farnesoid X receptor (FXR) and the membrane G protein-coupled receptor TGR5 (GPBAR1) play dominant roles in metabolic and immune regulation [93,94,95,96].

FXR signaling exerts anti-inflammatory effects by inhibiting NF-κB activity and reducing the expression of proinflammatory cytokines such as TNF-α, IL-6, and IL-1β. FXR also maintains intestinal barrier integrity by stabilizing tight junction proteins (claudin-1, occludin) and promoting angiopoietin-mediated antimicrobial peptide secretion. Additionally, BAs influence gut microbiota composition and activity, both directly and indirectly via receptor-mediated signaling and modulation of the intestinal environment [95,96,97].

TGR5 activation complements these effects by modulating inflammatory and immunoregulatory processes. It influences NLRP3 inflammasome activity, cellular metabolism, and regulatory T-cell differentiation. Through FXR and TGR5, BAs act on immune cells, including macrophages, dendritic cells, and lymphocytes, thereby promoting immune homeostasis and limiting chronic inflammation [93,96,97].

Within the gut–skin axis, bile acids influence skin cells such as keratinocytes, dermal dendritic cells, and macrophages by modulating pro- and anti-inflammatory cytokine expression. They also regulate transcription factors involved in immune control, including Foxp3, promoting Treg differentiation and suppressing proinflammatory Th17 responses through RORγt downregulation [98,99].

Secondary bile acids integrate metabolic and immunological signals that shape the skin’s inflammatory environment. Dysregulation of BA metabolism may promote pathological immune activation and contribute to systemic and cutaneous inflammation observed in chronic inflammatory dermatoses such as psoriasis and atopic dermatitis [98].

Together with SCFAs and tryptophan-derived metabolites, bile acids form a major microbiota-derived signaling network linking intestinal microbial activity with systemic immune regulation and skin homeostasis.

## 3. The Immunometabolic Amplifier Model: A Conceptual Framework of the Gut–Skin Axis

Gut dysbiosis may act as an immunometabolic amplifier of inflammatory processes in chronic skin diseases such as AD and psoriasis. This concept should be understood as a hypothesis-generating framework integrating current evidence on microbiota–host interactions rather than a fully experimentally validated mechanism. It refers to the ability of dysbiotic microbial communities to influence both metabolic signaling and immune regulation, thereby potentially amplifying inflammatory pathways along the gut–skin axis.

In this framework, dysbiosis is not only a consequence of systemic inflammation but may also act as an active driver that promotes a self-perpetuating proinflammatory loop between the gut microbiota, immune system, and peripheral tissues, including the skin. While bidirectional microbiota–immune interactions are well recognized, the existence of a unified amplification loop remains largely conceptual.

This amplification may occur through several interconnected mechanisms, including intestinal barrier dysfunction associated with dysbiosis, increased translocation of bacterial components, and alterations in the production of bioactive microbial metabolites such as SCFAs and tryptophan metabolites. These mechanisms are primarily supported by mechanistic studies, with growing but still limited evidence from human research.

Through these pathways, gut dysbiosis may modulate host immune responses and contribute to systemic inflammatory processes that also affect the skin. The following subsections outline key factors associated with gut dysbiosis and summarize mechanisms through which it may influence inflammatory pathways within the gut–skin axis [2,11,32].

### 3.1. Origins of Dysbiosis

Dysbiosis refers to an imbalance in the composition and functional activity of the gut microbiota. It includes both a reduction in beneficial microorganisms and an excessive expansion of pathogenic species, which leads to structural and functional alterations of the microbiome. It is often accompanied by a decrease in microbial diversity and disturbances in microbial metabolic activity. As a result, the homeostasis between the microbiota and the host is disrupted, which may lead to increased intestinal barrier permeability, dysregulation of the immune response, and the development of chronic inflammation [33,100,101,102,103].

Multiple factors contribute to its development, including genetic predisposition, diet, medications (especially antibiotics), and environmental influences. Persistent dysbiosis can trigger chronic or recurrent inflammatory diseases, often extending beyond the gut, and is observed in inflammatory skin conditions such as AD and psoriasis [25,100,101].

#### 3.1.1. Genetic Factors Shaping the Microbiome

Genetic factors shape gut microbiota composition and function, influencing susceptibility to microbiome-related disorders. Studies, including the TwinsUK cohort, show that monozygotic and dizygotic twins share microbiome-related host traits, such as body weight, blood pressure, protein secretion, and antibiotic resistance, with notable similarity among Bacillota (formerly Firmicutes) and Verrucomicrobia. Specific host genetic loci have also been shown to be significantly associated with the risk of developing microbiome-dependent diseases and with the modulation of interactions between the gut microbiome and the host. Among the best characterized are solute carrier family 22 member 5 (SLC22A5), G protein-coupled receptor 35 (GPR35), and GPR65, which are associated with the risk of developing inflammatory bowel disease (IBD) [100].

Variants of individual genes can directly modulate gut microbiota composition. For example, the lactase locus (LCT) associates with Acetobacter and Bifidobacterium abundance, while interactions between ABO and FUT2 variants influence specific bacterial groups by altering the intestinal environment [100,104].

#### 3.1.2. Dietary Patterns and Gut Microbiota

Dietary patterns are a key factor shaping gut microbiota and maintaining host-microbiota symbiosis. The gut microbiota performs metabolic functions such as digesting complex polysaccharides, producing SCFAs, metabolizing bile acids, and synthesizing vitamins, all essential for intestinal barrier integrity and immune modulation. Microbiota-accessible carbohydrates (MACs), mainly from dietary fiber, strongly influence colonic microbiota activity. Diets high in animal fats and proteins but low in fiber reduce *Bacillota*, increase bile-tolerant bacteria (*Alistipes*, *Bilophila*) and *Proteus*, and promote inflammation via LPS absorption, subclinical endotoxemia, and TLR4 activation. Low MAC intake reduces SCFA production, while high-fiber, low-fat diets favor an anti-inflammatory microbiome profile. These findings highlight diet’s major role in gut composition and inflammatory potential [100,101,105].

#### 3.1.3. Medications and Gut Microbiota

Medications significantly shape gut microbiota composition and stability, with antibiotics being the most impactful. They induce dysbiosis by disrupting microbial balance and reducing gut microbial diversity, often increasing Proteobacteria (including Enterobacteriaceae), which promotes proinflammatory states and antibiotic resistance [100,105]. Common antibiotics affecting the microbiota include vancomycin, ampicillin, streptomycin, and metronidazole, and their use can increase susceptibility to *Clostridioides difficile* colonization [106]. Recovery of the gut microbiota depends on host factors and the duration of antibiotic exposure [100,105].

Nonsteroidal anti-inflammatory drugs (NSAIDs) also significantly affect the gut microbiome. Widely used and often available over the counter, NSAIDs can disrupt microbial balance through direct effects on microorganisms and indirect effects related to mucosal damage and altered intestinal physiology. Conventional NSAIDs are associated with an increased risk of small intestinal injury [107].

Proton pump inhibitors (PPIs) are another commonly used drug class that can alter the gut microbiome. Their use has been linked to unfavorable changes in microbial composition, increasing susceptibility to *C. difficile* and other intestinal infections [108]. These effects are class-related: omeprazole, esomeprazole, and pantoprazole induce similar microbiome alterations, more pronounced at higher doses [109].

While these medications are essential for therapy, their potential impact on the gut microbiota should be carefully considered when evaluating indications for use [100,109].

#### 3.1.4. Early-Life Development, Environmental Influences, and Immune Imprinting

Environmental influences, including stress, infections, and hygiene, play a key role in shaping the gut microbiota and the development of dysbiosis. Chronic stress can modulate microbial composition and impair host physiological functions. Infections may disrupt microbial balance, while bacterial toxins can damage the intestinal barrier and promote proinflammatory states [100,110].

Social interactions and early-life exposures are particularly critical: vaginal birth and breastfeeding support beneficial gut colonization, whereas cesarean delivery, formula feeding, and highly hygienic environments can alter early gut microbial development. Colonization of the gut microbiome during infancy is crucial for immune system maturation and the process of immune imprinting, where interactions between the developing gut microbiota and the host immune system shape long-term immune function and tolerance [111,112].

Proper microbial exposure in early life promotes the development of regulatory immune pathways, including regulatory T cells (Treg), which support immune tolerance and help prevent excessive inflammatory responses [113].

Disruptions in early-life gut microbiome development may predispose individuals to immune-mediated diseases, particularly atopic dermatitis, by promoting immune dysregulation and impaired tolerance. While evidence for psoriasis is more limited, early intestinal microbial imbalances could similarly affect long-term immune responses, highlighting the potential role of early-life gut dysbiosis in triggering inflammatory pathways along the gut–skin axis [114].

### 3.2. Intestinal Barrier Dysfunction and Increased Permeability

#### 3.2.1. Zonulin-Mediated Tight Junction Disruption

Immunological homeostasis of the gut and the entire organism is maintained by the intestinal barrier, a complex system composed of mucus and the gut microbiome. Proteins within this barrier enable selective permeability for nutrients and protect against pathogens, toxins, and antigens [115]. Disruption of the intestinal barrier increases permeability, contributing to inflammatory diseases [116].

An important regulator of intestinal tight junctions (TJs) is zonulin, whose release is induced, among other factors, by gut dysbiosis [116,117]. Zonulin activates the epidermal growth factor receptor (EGFR) via protease-activated receptor 2 (PAR2), causing phosphorylation of TJ proteins and actin filament reorganization. This leads to TJ repression, increased permeability, and elevated levels of proinflammatory cytokines such as IL-12 and IFN-γ, promoting translocation of food residues and bacterial antigens and activating T lymphocytes. The main triggers of zonulin release are bacteria and gliadin [13,15].

It has been hypothesized that zonulin release may serve as a defensive mechanism, as increased intestinal permeability can prevent adhesion and colonization of pathogenic bacteria. Gliadin, a component of gluten, stimulates zonulin release via CXCR3 activation through a MyD88-dependent signaling pathway, further increasing permeability [116,118]. Dysregulation of the intestinal barrier and the resulting rise in inflammatory mediators can trigger systemic inflammation, contributing to disrupted skin homeostasis. Inflammatory skin diseases are linked to gut dysbiosis, partly through this mechanism of barrier dysfunction [25].

#### 3.2.2. Metabolic Endotoxemia and LPS Translocation

Metabolic endotoxemia involves elevated levels of circulating bacterial endotoxins, particularly lipopolysaccharide (LPS). LPS, a key component of Gram-negative bacterial outer membranes, is a potent activator of the inflammatory response and functions as a MAMP. Dysbiosis and increased intestinal permeability facilitate LPS translocation into the bloodstream [119,120,121].

Once in circulation, LPS activates TLR4, triggering the production of proinflammatory cytokines such as TNF-α, IL-6, and IL-1β. This promotes infiltration of activated macrophages into peripheral tissues, with further recruitment of neutrophils and monocytes, contributing to persistent inflammation and disruption of tissue homeostasis [120,121,122,123].

#### 3.2.3. Nutritional Malabsorption and Skin-Essential Micronutrients

Disorders of nutrient absorption impair uptake of micronutrients essential for skin function, including zinc, vitamins A and D, and omega-3 fatty acids, reducing epidermal regeneration capacity. Such deficiencies may result from inadequate dietary intake or impaired intestinal absorption due to infections or chronic inflammation [124].

Deficiencies of these key micronutrients have broad systemic consequences, notably affecting skin physiology due to their essential roles in maintaining skin health. Vitamin A and its derivatives (retinoids, carotenoids) regulate cellular proliferation, differentiation, and apoptosis in epithelial tissues. Vitamin D supports antimicrobial activity, modulates inflammation, and aids wound healing via cAMP pathways [125]. Omega-3 fatty acids contribute to skin regeneration, and zinc acts as a cofactor for metalloenzymes while protecting against UV-induced damage [125,126].

Micronutrients exert pleiotropic effects on skin function, including the stimulation of repair processes and wound healing. Dysbiosis that disrupts micronutrient metabolism and absorption may therefore limit these beneficial effects [127].

### 3.3. Systemic Immune Activation Induced by Gut Dysbiosis

Gut dysbiosis triggers a cascade of systemic immune events, linking innate sensing, hepatic responses, and microbial metabolite signaling to skin inflammation, thereby potentially contributing to downstream pathogenic mechanisms in chronic dermatological conditions.

#### 3.3.1. SCFA- and AhR-Mediated Signaling Pathways in Skin Immune Responses

While Section 2.2.1 outlines the role of microbiota-derived metabolites, the following section highlights the downstream signaling pathways through which SCFAs and AhR activation influence cutaneous immune responses.

SCFAs influence systemic and cutaneous immune responses through several downstream signaling mechanisms, including receptor-mediated pathways, epigenetic regulation, and modulation of cellular metabolic signaling.

The first, receptor-mediated pathway involves G protein-coupled receptors such as GPR41, GPR43, and GPR109A. Activation of these receptors stimulates the production of IL-22 by innate lymphoid cells type 3 (ILC3), enhances epithelial barrier integrity, and supports mucosal immune responses, including increased IgA production.

In the epigenetic pathway, SCFAs inhibit histone deacetylases (HDACs), which reduce NF-κB activation and decrease the production of proinflammatory cytokines. At the same time, this mechanism increases *FOXP3* expression, promoting the differentiation of regulatory T cells (Tregs) and the production of anti-inflammatory cytokines such as IL-10 and TGF-β.

Additionally, SCFAs modulate the mTOR signaling pathway, regulating the activation and differentiation of T lymphocytes, including Th1 and Th17 cells, thereby helping to maintain the balance between inflammatory responses and immune tolerance [70,128].

Microbial metabolites derived from tryptophan can activate the aryl hydrocarbon receptor (AhR), a ligand-dependent transcription factor that regulates cytokine production, including IL-22, and influences epithelial barrier integrity and epithelial cell responses, including those of keratinocytes [129]. After ligand binding in the cytoplasm, AhR translocates to the cell nucleus, where it forms the AhR-ARNT complex, which binds to DNA sequences known as xenobiotic response elements (XREs), leading to the activation of transcription of target genes. These include both immunoregulatory cytokines, such as IL-22, and genes associated with epidermal barrier function, including FLG and LOR. As a result, activation of the AhR signaling promotes keratinocyte differentiation and strengthens skin barrier integrity [86,130].

Both pathways contribute to maintaining immune homeostasis by regulating cytokine production and immune cell differentiation. Through these mechanisms, they contribute to the regulation of inflammatory processes along the gut–skin axis [131,132].

#### 3.3.2. Innate Immune Sensing and NLRP3 Inflammasome

During infection, the immune system recognizes microorganisms through pattern recognition receptors (PRRs), which detect pathogen-associated molecular patterns (PAMPs) and initiate inflammatory signaling pathways, including inflammasome activation. In the context of gut dysbiosis, microbial components such as lipopolysaccharide (LPS) and other microbe-associated molecular patterns can further stimulate PRRs, providing the priming signal required for the NOD-like receptor pyrin domain-containing 3 (NLRP3) inflammasome activation [133,134].

The inflammasome, a multiprotein complex, regulates mucosal metabolic activity and activates caspase-1, leading to proteolytic maturation of pro-IL-1β and pro-IL-18 into their active forms, key mediators of the proinflammatory response [133,135].

Activation typically requires two signals: a priming signal via PRR-mediated NF-κB activation (inducing NLRP3 and pro-IL-1β expression) and a second signal triggered by cellular stress or microbial stimuli, such as ATP or bacterial toxins [136].

The NLRP3 inflammasome thus represents a critical link between gut microbiota and systemic inflammation. Gut dysbiosis can promote NLRP3 activation, leading to recruitment of inflammatory cells and excessive cytokine production (IL-1β, IL-6, TNF-α), which may contribute to skin inflammation. IL-1β can directly affect keratinocytes, increasing IL-6 and TNF-α production and influencing skin barrier function and inflammatory responses [135,137,138].

#### 3.3.3. Gut–Liver–Skin Axis and Systemic Inflammation

The intestines, liver, and skin form an axis allowing for mutual influence. The intestines and liver communicate via portal circulation, bile ducts, and systemic circulation. Hepatocytes stimulated by gut-derived factors secrete acute-phase proteins, complement proteins, and other substances to control infection. Kupffer cells maintain immune balance by capturing and presenting bacterial antigens and participating in iron metabolism. Low exposure to gut-derived LPS promotes endotoxin tolerance, increases inhibitory factors such as PD-L1, and supports regulatory T-cell development with anti-inflammatory cytokine secretion (IL-10, TGF-β) [139].

In contrast, gut dysbiosis increases intestinal permeability, allowing endotoxins, bacterial metabolites, and host-derived products to reach the liver via the portal vein. This triggers hepatic production of proinflammatory cytokines (IL-1β, IL-6, TNF-α) and recruitment of neutrophils and monocytes [140,141].

Chronic inflammation caused by dysbiosis, amplified by hepatic immune cells, can extend to the skin. Animal studies show that systemic inflammation from gut dysbiosis disrupts the balance between effector and regulatory T lymphocytes, impairing tolerance to microbiota and promoting inflammation, which may compromise skin homeostasis [40].

#### 3.3.4. Microbial Metabolites and Skin Barrier Function

Dysbiosis is associated with various skin diseases, including acne vulgaris, atopic dermatitis, and psoriasis. However, it is often unclear whether dysbiosis contributes to disease development or results from the condition. Gut bacteria produce neurotransmitters and metabolites that can cross the intestinal barrier and enter the bloodstream, exerting systemic effects [11].

Phenolic compounds, such as p-cresol, a metabolite of aromatic amino acids produced by gut bacteria, are considered biomarkers of dysbiosis. Animal studies show that phenols accumulate in the skin and disrupt keratinocyte differentiation, while human studies indicate that elevated serum p-cresol reduces skin hydration, impairs barrier integrity, and negatively affects keratinization [25,142]. These findings suggest that microbial phenol production can contribute to intestinal and skin barrier dysfunction and impair keratinocyte maturation [11,142,143].

### 3.4. Immune-Mediated Skin Effects of Gut Dysbiosis

Gut dysbiosis drives systemic immune alterations that promote chronic skin inflammation through both cellular and molecular pathways. Building upon the mechanisms of T-cell education described in Section 2.1.3, it leads to an aberrant, pathological “homing” of gut-primed T lymphocytes to the skin, generating “dual-tropic” lymphocytes that traffic to both the gut and skin [60].

Among these, CLA^+^ T cells drive skin inflammation in conditions such as atopic dermatitis, psoriasis, and allergic reactions [144,145,146] by producing proinflammatory interleukins (IL-4, IL-5, IL-13) and stimulating B-cell IgE production, which affects eosinophil survival and promotes keratinocyte damage [145].

Systemic immune activation induced by dysbiosis alters barrier function, innate sensing, and adaptive immune responses, creating an environment that favors persistent skin inflammation. proinflammatory cytokines can suppress key epidermal structural proteins and antimicrobial peptides, compromising skin barrier integrity. Gut-derived metabolites, particularly short-chain fatty acids such as butyrate, may partially counteract these effects by supporting keratinocyte differentiation and barrier function through mitochondrial metabolism. The molecular and immunological mechanisms underlying disease-specific patterns are discussed in detail in Section 4 [5,147,148,149,150].

Chronic skin inflammation, a hallmark of many dermatological diseases, can in turn influence gut microbial composition, establishing a bidirectional feedback loop that may disrupt systemic immune balance. This highlights the less-studied reverse pathway in the gut–skin axis, linking gut dysbiosis to persistent cutaneous and systemic immune alterations [5,40].

## 4. Comparative Pathology: Atopic Dermatitis vs. Psoriasis

### 4.1. Immunological Landscapes and Immune Imprinting

#### 4.1.1. Immunopathogenesis of Atopic Dermatitis

The key and most characteristic element of the pathogenesis of atopic dermatitis (AD) is persistent polarization of the immune system toward a type 2 response and the dominance of the IL-4/IL-13 axis [151,152]. This process is strongly influenced by genetic predispositions and early-life events during the neonatal and infant period, which together shape immunological imprinting. In this context, complex genetic mechanisms compromise epidermal barrier integrity and regulate both innate and adaptive immune responses, highlighting the intricate molecular pathways underlying the disease [153].

Genetic studies provide strong evidence that mutations in the filaggrin (*FLG*) gene represent the most significant and well-documented factor contributing to barrier dysfunction. *FLG* is one of approximately 70 genes comprising the epidermal differentiation complex (EDC), a ~2 Mb region on chromosome 1q21 that is essential for keratinocyte maturation and the maintenance of barrier integrity [154,155,156,157]. Other barrier-associated genes within the EDC, including *FLG2*, *SPRR3*, *LOR*, and *HRNR*, also regulate epidermal function and have been linked to AD [157,158,159,160]. Beyond the EDC, the *SPINK5* gene further influences barrier integrity, with the Asn368Ser variant increasing susceptibility to disease [161].

These genetic variants additionally shape both innate and adaptive immune pathways, particularly those driving type 2 (Th2) immunity. Key examples include Th2-associated genes (IL-4, IL-13, IL-4RA, IL-13RA1, IL-13RA2, STAT6), genes encoding thymic stromal lymphopoietin (TSLP) and its receptors (IL-7R, TSLPR), genes involved in innate immunity (pattern recognition receptors [PRRs], antimicrobial peptides [AMPs]), vitamin D pathway genes, and genes related to the ligand-binding subunit of the high-affinity IgE receptor (FcεRI) [157,162,163].

During early-life immunological imprinting, these genetic factors influence the maturation and responsiveness of multiple immune components, including the epidermal barrier, skin microbiota colonization, and the establishment of specific cellular populations in the skin [164,165]. Even minor damage or leakiness of the epidermal barrier at this stage, leading to exposure of the deeper layers of the skin to antigens (e.g., microbial, toxin, or environmental allergens), initiates the secretion of signaling molecules from keratinocytes known as alarmins. These include factors such as TSLP, IL-33, and IL-25, which activate innate lymphoid type 2 cells (ILC2) and antigen-presenting cells (APCs), including dendritic cell subsets [153,166,167,168,169]. The effect of this stimulation is increased expression of IL-13 (and IL-5), which is responsible for promoting a type 2 response by shaping the tissue microenvironment [170]. A key element directly promoting the differentiation of naive CD4^+^ lymphocytes toward the Th2 phenotype is local IL-4 activity [151,171]. At this stage, the source of expression of this cytokine may be basophils, mast cells, and some other lymphocyte phenotypes.

A complement to the phenomenon of imprinting in AD is the development of mechanisms that consolidate the predominance of the Th2-dependent response. The processes described above, together with the Th2-lymphocyte response mediated by the IL-4/IL-13 axis (discussed further), induce persistent alterations in the skin’s immunological memory, driven primarily by the accumulation of tissue-resident memory T cells (TRM), which facilitate the rapid initiation of inflammatory cascades in defined skin regions [62]. In AD innate populations, such as ILC2, may also exhibit an increased baseline readiness to respond to alarmin signals [172].

After the establishment of imprinting toward a type 2 response, the main mechanism of AD pathogenesis is a positive feedback loop involving Th2 lymphocytes and the IL-4/IL-13 cytokines they secrete upon exposure to an AD-triggering factor [173,174]. At the molecular level, the most important element of pathogenesis is signaling mediated by receptors containing the transmembrane IL-4Rα subunit, which constitutes the binding site for IL-4 and participates in binding IL-13. Receptor complexes containing IL-4Rα include the Type I IL-4 receptor complex (Type I IL-4R), dedicated to IL-4, and the Type II IL-4 receptor complex (Type II IL-4R), which binds both IL-4 and IL-13 [175]. An additional binding site for IL-13 is Interleukin 13 Receptor Alpha 2 (IL-13Rα2), which lacks the IL-4Rα subunit and is classically regarded as a decoy receptor, thereby reducing IL-13 availability for Type II IL-4R [176].

Type I IL-4R is located on lymphocytes and myeloid cells [177]. Its stimulation leads to activation of the JAK1 and JAK3 kinases, which initiates a pathway resulting in phosphorylation and dimerization of the STAT6 transcription factor, which directs gene expression toward the type 2 response program [178,179]. The downstream functional consequences of Type I IL-4R stimulation include differentiation of naive CD4^+^ cells toward the Th2 phenotype, class switching of B-lymphocyte immunoglobulins toward IgE, and enhancement of mast cell functions [171,180].

Type II IL-4R is expressed on wide range non-hematopoietic cells—primarily keratinocytes and fibroblasts—although it is also present on immune cells [181]. Stimulation of this receptor complex leads to activation of the JAK1 and TYK2 kinases (with involvement of JAK2 also sometimes described), which, similarly to the Type I IL-4R signaling pathway, results in phosphorylation and dimerization of the STAT6 factor [181,182]. When considering keratinocytes, stimulation of this receptor is associated with decreased expression of filaggrin, loricrin, and involucrin—proteins considered as epidermal barrier-associated differentiation markers [138]. Processes of metabolism of other barrier components, as well as the process of epidermal differentiation, are also disrupted [138,183]. Via STAT6, there is also an increase in the expression of the chemokines CCL17 and CCL22, produced locally, inter alia, by keratinocytes and dendritic cells [184,185]. These factors are responsible for chemotaxis of cells expressing the CCR4 receptor on their surface (typically Th2 lymphocytes) [186]. In addition, expression of the chemokine CCL26 increases, which induces eosinophil influx [187,188]. The above signaling mechanisms mediated by Type I and Type II receptors for IL-4/IL-13, as well as the role of IL-13Rα2, are summarized in Table 1.

Knowing the mechanisms described above, a significant positive feedback loop becomes apparent. IL-4 and IL-13 secreted by Th2 lymphocytes stimulate Type I and Type II IL-4R. The overall consequence of this stimulation is polarization of naive CD4^+^ cells toward Th2 lymphocytes, increased chemotaxis of this phenotype into inflamed areas, and worsening of epidermal barrier dysfunction. This, in turn, increases the skin’s susceptibility to environmental factors and injury, promoting secondary amplification of epithelial signals (alarmins), enhanced activation of antigen-presenting cells, and consequently further IL-4 and IL-13 expression by Th2 lymphocytes.

Although the pathways described above are the most characteristic of AD, it should be added that, in some patients, other axes may also predominate, such as Th22/Tc22 or Th17/Tc17 [189]. A particular situation involving a relative shift toward Th1/Th17 in the dominant pathway is the chronic stage of AD [190].

Additionally, inflammatory processes in the skin may be exacerbated by excessive colonization with *Staphylococcus aureus*, which is a well-documented feature of AD [191]. Such alterations in the flora affect both lesional and non-lesional skin, although to a lesser extent in the latter case. It is currently considered a separate factor that amplifies the disease process in AD [192,193].

According to the current literature, the cutaneous environment, dominated by a Th2-dependent response and barrier dysfunction, promotes adhesion and persistence of S. aureus by increasing local pH, weakening antimicrobial mechanisms, and damaging the structural proteins of the barrier [194,195].

In turn, *S. aureus* intensifies inflammation at multiple levels (for example through the production of toxins, superantigens, and proteases) which further disrupts barrier integrity and increases the activation of cells involved in the type 2 response [193,195]. It is also important to emphasize the positive correlation between higher S. aureus burden and greater AD severity [191].

**Table 1:** Summary of information on IL-4 and IL-13 receptors from perspective of AD pathogenesis.

Feature	Type I IL-4R	Type II IL-4R	IL-13Rα2
Subunit composition	IL-4Rα + γc [176]	IL-4Rα + IL-13Rα1 [176]	IL-13Rα2 [176]
Ligands	IL-4 [175]	IL-4, IL-13 [175]	IL-13 [176]
Expressing cells (key from the perspective of AD)	Lymphocytes, myeloid cells; most importantly naive CD4^+^, B lymphocytes, mast-cells [177]	wide range non-hematopoietic cells (including keratinocytes, fibroblasts, immune cells) [181]	Both immune and non-immune cells, depending on the tissue [196,197]
Key signaling pathway	JAK1/JAK3 → STAT6 [178]	JAK1/TYK2/(±JAK2) → STAT6 [181,182]	Canonically a decoy-type receptor; no typical IL-4R/STAT6 pathway signaling [176]
Most important effect	Promotion of the Th2 phenotype, class switching toward IgE, enhancement of mast cell functions [171,180]	Disruption of production of epidermal barrier components and epidermal differentiation; chemotaxis of Th2 lymphocytes and eosinophils [138,183,186,187,188]	Reduced availability of IL-13 for Type II IL-4R [176]

SCFAs, primarily acetate, propionate, and butyrate, belong to the group of gut microbiota metabolites and are produced as a result of polysaccharide fermentation in the gastrointestinal tract [198]. Their availability has been linked to the development of immunological tolerance in early life and to the risk of atopic diseases, including AD [199,200]. The current literature highlights an association between low fecal SCFA concentrations in infants and a higher frequency of allergic manifestations later in life. Conversely, higher concentrations of propionate and butyrate in early childhood are associated with a lower risk of atopic diseases in cohort studies [201].

The shaping of tolerance by SCFAs is based on promoting and stabilizing populations of regulatory T lymphocytes expressing the forkhead box P3 (Foxp3) transcription factor (Foxp3^+^ Tregs). This promotion occurs through inhibition of histone deacetylases (HDACs), which increases histone acetylation in regions involved in regulating *FOXP3* expression, thereby increasing the production of this regulator [202,203]. In addition, HDAC inhibition also promotes acetylation of the Foxp3 protein itself, which is described as an aspect that increases its stability [204]. Foxp3 is a key transcription factor that determines the differentiation and stability of the Treg phenotype and regulates the expression of genes responsible for the suppressive function of these cells in the context of the immune response [205].

SCFA activity also involves acting as ligands for G protein-coupled receptors (GPCRs). This group includes Free Fatty Acid Receptor 2 (FFAR2), Free Fatty Acid Receptor 3 (FFAR3), and Hydroxycarboxylic Acid Receptor 2 (HCAR2). These GPCRs are expressed on the surface of many types of immune system cells, and their activation, in certain models, promotes immunological tolerance [206,207].

#### 4.1.2. Immunopathogenesis of Psoriasis

Just as in AD the central role in pathogenic mechanisms typically belongs to Th2 lymphocytes and the IL-4/IL-13 axis, in psoriasis the main role is played by Th17 lymphocytes and the IL-23/Th17/IL-17 axis [208,209]. In this case, immune imprinting is less dependent on phenomena in early childhood, and the most important factors are genetic and environmental predispositions, responsible for a greater propensity for an inflammatory response involving IL-23/IL-17, and for the occurrence of a triggering stimulus (e.g., microtrauma, infection, stress, medications, tobacco smoking, mental stress) [210,211].

Among the genetic factors associated with susceptibility to psoriasis, one of the strongest is the HLA-C*06:02 allele. In addition, deletion of the *LCE3B* and *LCE3C* genes, which encode late cornified envelope (LCE) proteins involved in epidermal barrier defense, constitutes a genetic factor that increases the risk of developing the disease. *LCE3* genes encode proteins that participate in skin barrier repair following injury or inflammation. Their expression has been shown to be strongly elevated in psoriatic lesions and can also be induced in normal epidermis after superficial skin damage. This suggests that variations in genes related to barrier repair may influence disease risk. However, unlike in atopic dermatitis, barrier dysfunction in psoriasis does not represent a primary pathogenic mechanism, as immune pathways, primarily the IL-23/Th17/IL-17 axis, play a dominant role [212,213,214,215].

In genetically and environmentally predisposed individuals, triggers cause mechanical damage or cellular stress in the local skin microenvironment, leading to the release of early inflammatory mediators by keratinocytes and other skin cells, primarily endogenous DNA/RNA from injured cells and cathelicidin (LL-37). In this situation, LL-37 forms a complex with DNA and/or RNA, which constitutes a strong signal stimulating the inflammatory response [216]. These complexes activate plasmocytoid dendritic cells through endosomal toll-like receptors (TLRs) promoting secretion of IFN-α and IFN-β [208]. IFN-α increases activation of antigen-presenting cells (including myeloid/inflammatory DCs and macrophages), that contributes to increase in APCs’ key inflammatory cytokines in psoriasis, especially TNF-α, IL-6, IL-12, and IL-23 [216,217]. The role of APCs also involves antigen presentation to lymphocytes, which under the influence of the previously released cytokines promote Th17 differentiation, activation and maintenance [218]. IL-23 is a key factor in above mentioned processes [208].

The next part of the pathogenesis is based on secretion of IL-17A, IL-17F, and IL-22 by Th17 and Tc17 lymphocytes (although production of these cytokines also occurs via other cells, e.g., Th22 lymphocytes, γδ T cells, or type 3 innate lymphoid cells) [219,220]. Both IL-17 family members bind to the heterodimeric IL-17RA/IL-17RC receptor on the surface of keratinocytes. As a result of activation of intracellular signaling pathways, a range of biologically active factors is secreted. Chemokines (e.g., CXCL1/CXCL2/CXCL8) are mainly responsible for attracting neutrophils. CCL20 drives chemotaxis of CCR6-expressing cells, most notably Th17 and Tc17 lymphocytes—an important component of the positive feedback mechanism in psoriasis [218,221]. In addition, an effect of IL-17A/IL-17F on keratinocytes is the secretion of a number of antimicrobial peptides and subsequent inflammatory proteins from these cells, e.g., β-defensins and S100A7/A8/A9 [222]. Via other factors secreted by keratinocytes (e.g., IL-36), dendritic cells are also activated and IL-23 production by myeloid cells is increased, which constitutes a complement to the positive feedback loop [223]. The literature also draws attention to a strong relationship between the IL-17 family and TNF-α and describes their actions as synergistic [224]. In turn, IL-22 affects keratinocytes via the IL-22R1/IL-10R2 receptor and causes their excessive proliferation and disturbances in epidermal differentiation, thereby accounting for the characteristic morphology of lesions in psoriasis [218].

After initiation of an extensive inflammatory reaction via pathways characteristic of psoriasis, imprinting is also finalized. Similarly to AD, after the immune response subsides, TRM cells remain within specific skin layers and are capable of rapidly re-initiating IL-17/IL-22 pathways [225,226].

It should also be noted that chronic activation of the Th17/Tc17/IL-23/IL-17 axis is not limited solely to the skin, and psoriasis should be considered a systemic disorder [227]. In some patients, features of systemic low-grade inflammation are observed, along with alterations in circulating inflammatory markers and mediators, which correlates with severity and risk of developing specific organ diseases [227,228]. A clinical reflection of this generalization includes, inter alia, psoriatic arthritis, as well as an increased prevalence of metabolic syndrome, obesity, insulin resistance/type 2 diabetes, dyslipidemia, hypertension, and cardiovascular diseases [227,229]. Shared immunological pathways, particularly those related to IL-23/IL-17, also link psoriasis with selected inflammatory diseases of other organs (e.g., inflammatory bowel diseases or uveitis), which further supports viewing it as a systemic disease [230,231].

In the context of psoriasis, SCFAs (acetate, propionate, butyrate) are considered metabolites of the gut–immunity axis that may modulate the disease-critical IL-23/Th17/IL-17 cascade [232]. The mechanisms responsible for this partially overlap with those observed in AD (promotion of regulatory responses and modulation of innate-cell activation). However, in psoriasis, their potential significance should be related primarily to the IL-23/Th17/Tc17/IL-17 axis and the myeloid–neutrophil component. At the receptor level, SCFAs act via the GPCRs FFAR2, FFAR3, and HCAR2 present on myeloid cells, affecting their activation and secretory profile [78,233,234,235]. In parallel, SCFAs may act independently of receptors as HDAC inhibitors, which favors epigenetic stabilization of a regulatory profile (inter alia, by consolidating *FOXP3* expression) and may shift the Treg/Th17 balance toward control of inflammation [203]. At the same time, it should be emphasized that the effects of SCFAs are dependent on the cell type and the dominant receptor; for example, FFAR2 signaling in neutrophils may support chemotaxis and granulocyte reactivity, which under certain conditions does not necessarily have an exclusively tolerogenic effect [78,236,237]. For this reason, observations regarding SCFAs or the contribution of SCFA-producing bacteria in psoriasis should be interpreted cautiously, treating SCFAs as an element of immunometabolic modulation rather than a single, unambiguously protective factor [238].

#### 4.1.3. Oxidative Stress and Redox Signaling in Disease Progression

Oxidative stress is defined as a transient or chronic imbalance between the excessive production of reactive oxygen and nitrogen species (ROS and RNS) and the impaired ability of the body to neutralize them through antioxidant systems. The skin is a source of free radicals which, at low concentrations, participate in defense against microorganisms and in cell differentiation. Excessive ROS levels lead to DNA modification, protein degradation, lipid oxidation, cell apoptosis, and tissue damage, thereby disrupting the function of T helper cells and other components of the immune response. Elevated cytokine levels increase ROS production, further weakening antioxidant mechanisms and establishing a self-perpetuating cycle between chronic inflammation and redox imbalance in psoriasis and atopic dermatitis [239,240,241].

In the acute phase of AD, cytokines secreted by Th2 lymphocytes, such as IL-4 and IL-13, activate the JAK/STAT, MAPK (p38, ERK), and NF-κB signaling pathways, leading to increased production of reactive oxygen species (ROS). Excessive ROS levels cause direct damage to cell membranes, resulting in disruption of the skin barrier, while simultaneously enhancing the expression of proinflammatory cytokines and further exacerbating skin inflammation. Furthermore, ROS stimulate additional release of proinflammatory cytokines, including IL-4, IL-13, IL-22, and IL-31, which sustain chronic inflammation [8,242].

In psoriasis, increased oxidative stress activates Th1 and Th17 cells as well as keratinocytes via the MAPK, NF-κB, and JAK/STAT pathways. This leads to the release of a broad spectrum of cytokines, including IL-17, IL-22, IL-23, and TNF-α from Th17 cells, as well as TNF-α, IL-6, IL-8, and antimicrobial peptides produced by keratinocytes. These mechanisms drive keratinocyte hyperproliferation, neutrophil recruitment, angiogenesis, and persistent skin inflammation [243,244,245].

In both AD and psoriasis, oxidative stress and impaired antioxidant defense mechanisms play a significant molecular role, leading to damage to proteins, lipids, and DNA. These processes exacerbate chronic skin inflammation and may interact with other pathogenic pathways. Although the mechanisms linking oxidative stress to the gut microbiome are not yet fully understood and conclusive evidence is lacking, it has been suggested that microbiome–host interactions within the gut–skin axis may modulate local and systemic redox states, thereby amplifying inflammatory responses and contributing to the development of inflammatory skin diseases [241,246].

### 4.2. Microbiota Signatures and Clinical Correlations

#### 4.2.1. Atopic Dermatitis Gut Microbial Profile

AD and psoriasis are increasingly described in the context of alterations in the gut microbiota [238,247]. When considering AD, these changes are often described using the concepts of α-diversity and β-diversity. The former refers to richness (the number of taxa) and evenness (the relative contribution of individual taxa) of the microbiota components within a single sample. When we speak of a decrease in α-diversity, this means that the sample exhibits reduced species diversity, or that one or several organism groups become dominant, displacing the remainder. β-diversity describes how the composition of the bacterial microbiota differs between samples—most often referring to comparison of an individual’s material with that of a control group [248].

The most frequently described gut microbiological profile in AD suggests a decrease in α-diversity in the pediatric population and a distinct β-diversity pattern compared with healthy individuals in most age groups [249,250]. Caution should be exercised in a pooled analysis of the literature on gut microbiota α-diversity in patients with AD, because the results of specific analyses differ substantially, particularly in adults [251]. The literature even provides examples of studies in which α-diversity positively correlated with the severity of AD [252]. Differences in results may arise from variables such as patient age, diet, prior antibiotic therapy, or differences in the choice of α-diversity index. However, regardless of changes in α-diversity, significant β-diversity alterations in patients with AD are emphasized [250]. In the absence of significant fluctuations in α-diversity, this most often indicates a compositional shift in the microbiota without loss of its complexity. It should be emphasized that the observed differences in α- and β-diversity depend to a significant extent on the analytical method used, which may influence the conclusions drawn [253].

In some studies, particularly in pediatric populations, significant changes in the gut microbiota are observed in the form of an increased relative abundance of the Enterobacteriaceae family (most commonly bacteria of the genera Escherichia, Enterobacter, and Klebsiella are reported) [254]. Reviews emphasize that, in adults, the results are more heterogeneous, which possibly may also reflect differences in patient age, diet, prior antibiotic therapy, as well as comorbidities [251]. In this context, inflammation is highlighted as an important factor promoting the expansion of Gram-negative enteric rods. Such conditions increase local oxidative stress and oxygen availability, thereby conferring a metabolic advantage to facultative anaerobes, such as Enterobacteriaceae [255]. An increased abundance of Enterobacteriaceae is also linked in the literature to greater severity of AD symptoms, including pruritus, via pathogen-associated molecular patterns (PAMPs) released into the circulation, such as lipopolysaccharide (LPS), which induce immune responses. Experimental models suggest that exacerbation of pruritus occurs through stimulation of Toll-like receptor 4 (TLR4) by LPS. It should also be noted that AD itself may promote this phenomenon by damaging the intestinal barrier and increasing absorption of PAMPs, for example via IL-4 and IL-13, which are characteristic of this disorder [256,257].

Many publications also emphasize a reduced abundance of tolerogenic microorganisms and decreased production of anti-inflammatory metabolites (including SCFAs) in the course of AD [251]. Across clinical and review studies, decreases in Lactobacilli and Bifidobacteria are repeatedly reported in AD cohorts. These two genera are also discussed as relevant for immune regulation early in life because they can promote anti-inflammatory cytokine production via regulatory T-cell stimulation [258]. In parallel, several cohorts report reduced abundance of butyrate-associated genera such as Faecalibacterium and Roseburia in AD compared with controls [259]. As a result of reduced SCFA production and absorption, disruption of intestinal barrier integrity occurs, along with attenuation of the previously described mechanisms related to HDAC inhibition and signaling via specific GPCRs [251,260]. The consequence of this may be an imbalance in the Foxp3^+^ Treg/Th2 equilibrium, as well as disorder of other pathways that lead to immunological tolerance.

In the available studies, the relationship between AD severity assessed using the SCORing Atopic Dermatitis (SCORAD) scale and gut dysbiosis is most often described as a taxonomic–functional association rather than solely a “diversity-based” association. This means that SCORAD may be linked both to taxonomic composition (relative enrichment or depletion of specific bacterial groups) and to the functional consequences of these shifts (gene/metabolite profiles and intestinal barrier features) [261,262]. In pediatric populations, it has been shown, that the relative abundance of Streptococcus in the gut microbiota positively correlates with SCORAD, whereas the abundance of Clostridium negatively correlates with SCORAD. In an infant cohort, the authors identified a gut pattern in which Streptococcus predominance co-occurred with a lower abundance of Clostridium. This pattern was associated with greater clinical severity (higher SCORAD) and a higher likelihood of persistence of AD symptoms. At the same time, the authors indicated that this microbiological profile co-occurred with differences in the functional potential of the early microbiome (i.e., in the set of genes/metabolic pathways), suggesting that disease severity may result not only from the presence of specific taxa, but also from which metabolic functions predominate in the gut [261]. In adults, findings regarding taxa alone are more heterogeneous [251]. However, more recent studies show more consistent correlations between SCORAD and compositional–metabolic markers of the gut–skin axis, i.e., a combination of microbiome shifts with gut-derived metabolites and indices suggesting intestinal barrier dysfunction/exposure to bacterial products (e.g., LBP), as well as with selected metabolites (e.g., indoxyl metabolites, SCFAs) that correlate with clinical activity [262].

#### 4.2.2. Psoriasis Gut Microbial Profile

The literature increasingly emphasizes the importance of the gut–skin axis as a factor influencing the course of psoriasis [247]. Many studies indicate disturbances involving two major bacterial phyla: Bacillota and Bacteroidota (formerly referred to as Firmicutes and Bacteroidetes). Some studies have described marked shifts toward an increased relative abundance of Bacillota and a decreased relative abundance of Bacteroidota in the gut microbiota structure [238]. On this basis, authors began to distinguish the Bacillota/Bacteroidota ratio (denoted as F/B due to the now-outdated nomenclature) [238]. Clinical studies report conflicting findings on F/B ratio alterations in psoriasis, and discrepancies may stem from heterogeneous cohorts and study design limitations [247]. Thus, despite its frequent use, the F/B ratio has not emerged as a consistent microbiome signature of psoriasis across studies.

Concurrently, many reviews highlight reduced α-diversity of the gut microbiota in psoriasis; however, many studies in this setting also report predominantly β-diversity shifts without significant changes in α-diversity [238]. Functionally, the most important aspect in psoriasis appears to be a reduced abundance of SCFA-producing bacteria with immunomodulatory effects and protective roles with respect to the intestinal barrier [263]. Mechanistically, the significance of gut dysbiosis in psoriasis is most often considered through the lens of its impact on the Treg/Th17 balance and the IL-23/Th17 axis, which is key to psoriasis pathogenesis [238]. Loss of SCFA producers and impairment of the intestinal barrier may limit signals that favor the induction and stabilization of Treg cells (and thus immunological tolerance), while simultaneously facilitating the maintenance of an environment that promotes proinflammatory responses [263]. This type of microbiota disturbance is also strongly dependent on the characteristics of the studied population [238].

Clinically, many studies have begun to advance the thesis of a correlation between the severity of gut dysbiosis and psoriasis activity, as measured by the Psoriasis Area and Severity Index (PASI) scale [264,265]. However, findings regarding these relationships are sensitive to the heterogeneity of study groups. When interpreting these correlations, it should be remembered that metabolic syndrome frequently co-occurs in psoriasis, which can substantially influence both the extent of gut dysbiosis and the clinical course of the disease [266,267,268]. Therefore, several studies adjust analysis by metabolic factors (e.g., through BMI, dyslipidemia, or insulin resistance) [269,270,271]. In some cohorts, positive associations of PASI with selected taxa (e.g., Faecalibacterium/Parabacteroides/Bacillota) were demonstrated, with no association between the F/B ratio and PASI [264]. In contrast, more consistently, positive correlations of PASI with markers of intestinal barrier damage and with the metabolic component (e.g., BMI) were observed [271].

### 4.3. Gut–Skin Molecular Mechanisms

In Section 4.3, issues related to the impact of the gut–skin axis on the pathogenesis of AD and psoriasis, respectively, were summarized, excluding the previously described mechanisms pertaining to SCFA-mediated induction of immunological tolerance.

#### 4.3.1. Gut-Atopic Dermatitis Axis and Barrier Modulation

In the case of atopic dermatitis, one of the main consequences of dysbiosis is exacerbation of barrier dysfunction—both in the gastrointestinal tract and in the skin [272]. One of the best-documented links connecting intestinal function with the clinical phenotype in AD is SCFAs [64]. Similarly to the promotion of Foxp3^+^ Treg populations, SCFAs, acting as HDAC inhibitors, may increase the expression of genes associated with differentiation and formation of the epidermal barrier, e.g., by increasing the expression of filaggrin, loricrin, and involucrin—key proteins of this barrier [66,273,274]. In the case of SCFA deficiency resulting from dysbiosis, epigenetic conditions arise that favor consolidation of a phenotype prone to barrier disruption, which translates into an increased risk of exposing the immune system to triggering factors [64,66].

Beyond SCFAs, an important element of the gut–skin axis in AD is the pathway related to bacterial tryptophan metabolism [275]. A properly structured microbiota generates ligands (e.g., indoles) that activate the intracellular aryl hydrocarbon receptor (AhR), which is associated with a positive effect on epidermal differentiation and the expression of barrier proteins [193]. At the same time, activation of the AhR receptor can modulate the inflammatory response through diverse effects on multiple cells participating in AD pathogenesis, including keratinocytes, dendritic cells, lymphocytes, ILC2, and mast cells [188,276,277,278,279]. The immunological effects of this modulation may vary depending on the target cell and the ligand involved [277]. A reduced abundance of bacteria metabolizing tryptophan into AhR ligands within the microbiota structure may impair the above-mentioned mechanisms, which in most publications is regarded as an unfavorable factor in the course of AD [193]. It should also be noted that ligands for the AhR receptor also include compounds derived from the diet or the environment, and the nature of the systemic effect following receptor activation may depend on the type of ligand. The literature provides examples of ligands that promote AD pathogenesis by intensifying inflammatory processes [280].

An important link between gut microbiota dysbiosis and the clinical phenotype of AD also involves the consequences of increased intestinal barrier permeability [128]. A reduced abundance of SCFA-producing bacteria and a shift toward other taxa may contribute to a proinflammatory milieu, impaired intestinal tight junction integrity, and increased absorption of bacteria-derived factors [128]. Consequently, PAMPs can more readily enter the circulation and, via pattern recognition receptors (PRRs), may exacerbate inflammation and/or lower the threshold of skin reactivity to triggering factors [256,281]. Accordingly, intestinal barrier disturbances promote the development of inflammatory processes that also disrupt the structure and function of the epidermal barrier, as described in detail in the section on the immunological landscape of AD [128].

#### 4.3.2. Gut–Psoriasis Axis and Hyperproliferation Modulation

In the gut–psoriasis relationship, beyond the previously discussed mechanisms of SCFA effects on immunotolerance, increasing attention is being paid to the influence of other classes of metabolites and of intestinal barrier dysfunction on the course of the disease [282,283]. In this case, similarly to AD, a gut not affected by dysbiosis may act as a source of bacterial metabolites that potentially modulate symptom severity, and as a barrier limiting exposure to factors that exacerbate inflammatory responses and hyperproliferation [238].

A well-documented factor influencing hyperproliferation in psoriasis is activation of the AhR receptor, through which keratinocyte gene expression is altered. As a result, AhR signaling regulates the production level and protein profile of the epidermal barrier (including keratins, filaggrin, loricrin, and involucrin) and normalizes keratinocyte differentiation [131]. There are also studies supporting that AhR activation exerts an inhibitory effect on the signaling pathway initiated by IL-22, which may reduce the hyperproliferative effect [284]. In an animal model with keratinocyte-specific AhR deficiency, hyperproliferation-associated stress keratins are markedly increased [285].

Similarly to AD, activation of AhR also results in modulation of immune cell functions [86]. In this case as well, the effect of activation may be either beneficial or detrimental, depending on the target cell and on the type of ligand responsible for activation [286]. The literature describes decreases in IL-17A/IL-17F, IL-22, and IL-23 levels, as well as reduced differentiation and/or activity of Th17 lymphocytes, in studies investigating therapeutic activation of the AhR receptor [130,287]. The opposite effect is highlighted by recent studies on the impact of indoxyl sulfate (I3S) on the course of psoriasis. According to the cited model, I3S produced with the involvement of the gut microbiota enhances the Th17-dependent response via AhR activation, thereby exacerbating disease symptoms, including hyperproliferation. This effect may be mediated by increased expression of IL-17A/IL-17F genes and of gene regions encoding IL-21 and IL-23 receptors in Th17 lymphocytes [282].

Another class of metabolites that may influence the course of psoriasis are bile acids [288]. The most important group of compounds are secondary bile acids (formed as a result of microbiota-mediated modifications), such as deoxycholic acid (DCA) and lithocholic acid (LCA), as well as their downstream derivatives. Their effects are based on multilevel modulation of the immune response [289]. They can directly affect the differentiation and function of Th17 lymphocytes, disrupt cytokine signaling pathways, and counteract Th17 lymphocyte chemotaxis [99,288]. Some studies also suggest that LCA derivatives (e.g., isoalloLCA) have the capacity to promote the Treg phenotype [290]. All of the above mechanisms may contribute to limiting the Th17-dependent response and, secondarily, to alleviation of symptoms, including epidermal hyperproliferation.

Table 2 provides a brief summary of the key information presented in Section 4.

## 5. Microbiota-Targeted Therapeutic Strategies

### 5.1. Pro-, Pre-, and Synbiotics

Probiotics, prebiotics, and synbiotics are recognized as important modulators of the gut microbiota. Probiotics are live microorganisms that, when administered in adequate amounts, confer a health benefit on the host. Commonly studied probiotic genera include members of the former genus *Lactobacillus*, *Bifidobacterium*, and *Streptococcus*, as well as the yeast *Saccharomyces boulardii.* Certain probiotic strains can produce biologically active substances, including lactic acid, hydrogen peroxide, and bacteriocins [291,292].

Probiotics have been investigated as adjunctive interventions in atopic dermatitis (AD), primarily through modulation of the Th1/Th2 balance, primarily through suppression of Th2-associated responses, which may reduce Th2-associated cytokines such as IL-4, IL-5, and IL-13 [291,292]. Effects on IFN-γ are strain-specific. Probiotics also inhibit pathogenic bacteria and may support skin barrier integrity [291]. In psoriasis, probiotic supplementation has been associated with increased IL-10, contributing to attenuation of excessive inflammatory responses [293].

Prebiotics are selectively utilized substrates that promote the growth and activity of beneficial microorganisms, including non-digestible fibers and oligosaccharides such as inulin and fructooligosaccharides. Prebiotic fermentation produces SCFAs (acetate, propionate, butyrate), which modulate immune responses, reduce proinflammatory cytokines, and maintain epithelial barrier integrity [292].

Synbiotics combine probiotics and prebiotics to enhance microbial survival, colonization, and functional activity [294].

Clinical studies of pro-, pre-, and synbiotics, both single- and multi-strain, report heterogeneous results. Most studies focus on *Lactobacillus* and *Bifidobacterium*, with outcomes assessed using SCORAD in AD and PASI or DLQI in chronic inflammatory skin diseases. Meta-analyses suggest that probiotics may reduce SCORAD in pediatric AD, although heterogeneity limits precise effect estimation. Improvements in PASI and DLQI have also been observed, indicating potential benefits as adjunctive therapies. These findings indicate that microbiota-targeted interventions may represent a promising adjunct to conventional therapies for chronic inflammatory skin diseases, although further well-controlled trials are needed [291,293,295,296,297,298].

### 5.2. Precision Nutrition

Precision nutrition may represent a supportive strategy in the management of skin diseases through modulation of the gut microbiota. A diet rich in dietary fiber and polyphenols may exert beneficial effects on the gut microbiome and, consequently, indirectly influence skin health. Both polyphenols and fiber-rich diets promote microbial production of SCFAs, which contribute to attenuation of inflammatory processes by decreasing proinflammatory cytokines and enhancing anti-inflammatory pathways. Patients with AD have been reported to exhibit reduced SCFA levels in fecal samples; therefore, dietary interventions enriched in fiber and polyphenols may provide supportive benefits in AD and psoriasis [64,299,300,301].

### 5.3. Fecal Microbiota Transplantation (FMT)

Fecal microbiota transplantation (FMT) involves introducing processed stool from a healthy donor into a recipient to directly modify the gut microbiota and achieve therapeutic benefits. FMT is established as safe and effective for recurrent *Clostridioides difficile* infections, and its potential use is expanding to extraintestinal conditions, including autoimmune and allergic diseases.

FMT may restore intestinal microbial balance, inhibit pathogenic bacteria, and increase short-chain fatty acids (SCFAs), supporting epithelial barrier integrity and limiting inflammation. Preclinical studies in mice and dogs with atopic dermatitis reported restoration of gut microbiota composition, re-established Th1/Th2 balance, reduced inflammatory cytokines, and improved skin outcomes.

In humans, FMT has been associated with improvements in eczema severity indices, such as EASI, and case reports suggest potential benefits in psoriasis, including PASI and DLQI. These findings indicate that FMT may represent a supportive adjunctive therapy for chronic inflammatory skin diseases, although further well-controlled trials are needed [302,303,304,305].

### 5.4. Postbiotics and Next-Generation Microbiome-Based Therapies

Postbiotics are preparations of inanimate microorganisms and/or their components that confer health benefits. They include SCFAs, exopolysaccharides, bacteriocins, antioxidant enzymes, surface layer proteins, indole derivatives, and bacterial lysates. These compounds strengthen epithelial barrier function and modulate immune responses [306]. Sodium butyrate, a widely studied postbiotic, exhibits anti-inflammatory properties and supports intestinal barrier integrity [307].

Indoles, produced by microbial tryptophan metabolism, act as ligands for the aryl hydrocarbon receptor (AhR), which plays a key role in regulating immune responses and maintaining epithelial barrier function. AhR activation promotes IL-22 production, antimicrobial peptide secretion, and regulatory pathways including IL-10 signaling, contributing to suppression of inflammatory responses and stabilization of tight junctions. Cutaneous AhR signaling has been implicated in immune axes relevant to inflammatory dermatoses, including IL-17/IL-23 in psoriasis and IL-4/IL-13 in atopic dermatitis. Orally administered indole derivatives may therefore represent an important mechanistic link within the gut–skin axis, though most clinical studies have focused on local receptor modulation [130,308,309,310].

Next-generation microbiome-based interventions include engineered bacteria capable of detecting disease-related signals and releasing therapeutic molecules at target sites. These microorganisms can also modulate host metabolic pathways, including SCFA production, thereby influencing immune responses and reducing inflammation. Preclinical studies, including examples such as engineered *Escherichia coli* Nissle 1917 (EcN), producing IL-2, have demonstrated their ability to restore immune balance and modulate inflammatory pathways. Given the established relationship between gut microbiota homeostasis and systemic immune regulation, these precision microbial therapeutics may represent a promising adjunctive strategy for chronic inflammatory skin disorders [311,312].

### 5.5. The Role of Antioxidant Supplementation in Therapeutic Strategies

Oxidative stress is an important factor in the pathogenesis of chronic inflammatory skin diseases, including atopic dermatitis (AD) and psoriasis. Antioxidant supplementation may be considered an adjunctive therapy aimed at modulating inflammatory responses [313,314]. Dietary antioxidants include vitamin C and vitamin E, polyphenols, flavonoids, and carotenoids. These compounds participate in the neutralization of reactive oxygen species (ROS) and support the maintenance of immune homeostasis [315].

In addition to these exogenous antioxidants, the body possesses intrinsic antioxidant defense mechanisms. The Kelch-like erythroid cell-derived protein with cap’n’collar homology-associated protein 1 (KEAP1)–nuclear factor erythroid-2-related factor 2 (NRF2) system coordinates antioxidant and anti-inflammatory defenses in the skin by inducing detoxification enzymes and suppressing proinflammatory cytokines. This mechanism supports redox homeostasis and essential skin functions, including epidermal differentiation and immune regulation, thereby potentially mitigating inflammation in atopic dermatitis (AD) and psoriasis. As such, the NRF2 pathway represents a promising therapeutic target for inflammatory skin diseases [316,317].

Moreover, some antioxidants may indirectly influence the gut–skin axis by modulating gut microbiota composition and microbial metabolite production, which in turn can affect skin inflammation and barrier function [318]. Due to their anti-inflammatory properties, antioxidants may contribute to systemic anti-inflammatory effects [315,318].

### 5.6. Clinical Perspectives on Microbiota-Targeted Interventions

Interest in microbiota-targeted approaches, including probiotics, prebiotics, synbiotics, postbiotics, and dietary interventions, has been growing as potential adjunctive therapies for atopic dermatitis and psoriasis, supported by an increasing number of clinical studies [292,293,296,319]. Table 3 provides a concise overview of selected studies, highlighting the intervention type, study population, and key clinical outcomes.

Despite these encouraging findings, the clinical evidence remains heterogeneous and limited. Many studies involve relatively small cohorts, short intervention periods, or strain-specific effects, which complicates direct comparison and generalization. Moreover, some studies have reported inconsistent or modest clinical benefits, as well as neutral or negative findings, highlighting variability in therapeutic responses [291,292,296].

In some cases, no statistically significant improvement in clinical indices such as SCORAD or PASI was observed compared with placebo, suggesting that the effectiveness of these interventions may depend on multiple factors, including the probiotic strain used, duration of therapy, and characteristics of the studied population. Therefore, although microbiota-targeted strategies appear promising as adjunctive approaches, their clinical efficacy and long-term benefits still require clear confirmation. Larger, well-designed randomized controlled trials are needed to clarify their therapeutic potential and translational relevance [292,293,296,319].

### 5.7. Bidirectional Interactions Between Skin Inflammation and the Gut Microbiome

The gut–skin axis is increasingly recognized as bidirectional. While disturbances in the gut microbiota may contribute to the development of skin inflammation, it has also been proposed that effective treatment of dermatological diseases may indirectly influence the intestinal environment. In inflammatory skin diseases such as AD and psoriasis, therapies targeting Th1, Th2, and Th17 immune pathways may help restore immune balance, potentially leading to reduced systemic inflammation and partial normalization of immune responses [11,32].

In psoriasis, biologic therapies have been reported to induce changes in gut microbiome composition following successful treatment [330]. Similar observations have been described in patients with AD, where biologic therapies targeting the Th2 pathway were associated with alterations in gut microbiota diversity and composition alongside clinical improvement [331].

However, the currently available evidence remains limited, and further studies are required to determine to what extent effective treatment of skin diseases may contribute to restoring gut microbiota balance [332].

## 6. Translational Relevance and Future Directions

Growing numbers of publications report numerous, heterogeneous changes in the gut microbiota of patients with chronic inflammatory skin diseases such as AD and psoriasis. However, merely observing these alterations does not determine their clinical utility. To harness their translational potential, efforts should focus on intensifying the development of standardized analytical and predictive models of gut microbiota composition in relation to the diagnosis, treatment, and monitoring of these disease entities. Achieving practical translation into clinical practice in the coming years will require integration of data from pharmacomicrobiomics, individual patient multi-omics profiles, and personalized medicine approaches. Such data consolidation may help identify mechanisms of therapeutic failure, recognize patterns associated with disease activity, and select and predict response to treatment [2,5,333,334,335,336].

Particularly promising is the use of metabolomics to develop biomarker panels based on gut microbiota-derived metabolites, including SCFAs and indoles. For instance, serum levels of the indole metabolite indoxyl sulfate have been shown to correlate with disease severity in psoriasis patients, supporting the concept that microbiota-derived metabolites may serve as non-invasive indicators of disease activity and predictors of disease course, thereby bringing gut microbiome research closer to clinical application [262,282].

Future studies should also broaden the perspective of the gut–skin axis to include the mycobiome and virome (including phages), which may affect the host immune system both directly, e.g., by stimulation or modulation of the intestinal barrier, and indirectly, by reshaping the bacterial ecosystem, enabling gene transfer, and altering the profile and bioavailability of metabolites. Recent metagenomic analyses have identified distinct gut fungal signatures in psoriasis, suggesting that mycobiome components may serve as microbial biomarkers and modulators of systemic immune pathways. Verification of these hypotheses requires carefully designed, standardized studies that account for both the diversity and functional–metabolic components [337,338,339,340].

In parallel, with the growing interest in microbiota-modifying interventions, it is necessary to systematically investigate long-term safety, particularly because the microbiome is a dynamic system and its durable modification may have effects extending beyond the skin. Equally important is the development of management algorithms in which dietary interventions and microbiota-targeted therapies may have a meaningful impact as adjunctive treatment [1,341,342,343,344].

Progress in this field is often constrained by methodological challenges. Further research should emphasize standardization in aspects such as controlling confounding factors, harmonizing sampling procedures, selecting analytical methods, and demonstrating causality. Particular attention should be paid to the substantial heterogeneity of studies conducted to date, including differences in patient cohorts, disease phenotypes, and methods of sample collection. Differences also arise from the choice of sequencing strategy, such as 16S rRNA sequencing or shotgun metagenomics, which differ in taxonomic resolution and functional insight. Moreover, confounding factors such as diet, medications used, and prior antibiotic exposure should also be taken into account in a more consistent manner, as they may significantly affect microbiome composition and hinder the drawing of conclusions relevant to a given disease entity. Such measures will increase the likelihood of generating results that go beyond simple correlations and may have a real impact on clinical management [238,247,251,345,346,347,348].

## 7. Conclusions

**Figure 3:**
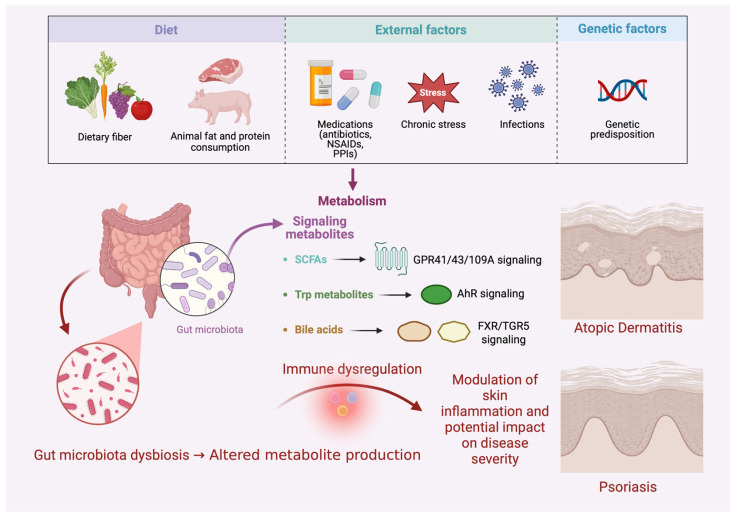
Integrated framework of gut–skin axis: interplay between contributing factors, altered microbial metabolites, immune signaling pathways, and their potential impact on inflammatory skin conditions. Created in BioRender. Andrzejczak, K. (2026) https://BioRender.com/4or862h.

Chronic inflammatory skin diseases, such as atopic dermatitis (AD) and psoriasis, exemplify the systemic nature of immune-mediated disorders, with skin pathology closely linked to gut microbiome composition and function. The gut–skin axis, a bidirectional network linking gut microorganisms, their metabolites, and host immunity, is increasingly recognized as a contributor to disease-specific mechanisms, including Th2/IL-4/IL-13-mediated barrier dysfunction in AD and Th17/IL-23/IL-17-driven hyperproliferation in psoriasis. Dysbiosis, reduced α-diversity, and diminished production of microbial metabolites, notably short-chain fatty acids and tryptophan-derived AhR ligands, may disrupt immune homeostasis and reinforce feedback loops that sustain chronic inflammation. The key mechanisms linking gut dysbiosis, microbiota-derived metabolites, immune signaling pathways, and skin inflammation are summarized in the graphical overview presented in Figure 3.

Microbiota-targeted interventions, including probiotics, synbiotics, fecal microbiota transplantation, and precision nutrition, show potential as adjunctive therapies by restoring microbial balance and promoting regulatory T-cell function. Their efficacy, however, is influenced by factors such as patient age, baseline microbiota composition, and the presence of dysbiosis. Importantly, most available evidence in humans remains observational and heterogeneous, limiting causal inference and the generalizability of findings.

Future research should integrate multi-omics analyses and functional profiling to identify predictive biomarkers and expand investigations to the mycobiome and virome to clarify their role in immune modulation. Harnessing gut–skin interactions represents a promising but still evolving framework to support AD and psoriasis treatment. When used alongside targeted therapies, this approach may help mitigate disease progression and improve outcomes. Nevertheless, it must be emphasized that these concepts remain largely theoretical and require well-designed clinical studies to validate efficacy and optimize therapeutic protocols. Overall, the gut–skin axis constitutes a biologically supported but not yet fully validated framework. While microbiota alterations are consistently associated with AD and psoriasis, causality in human studies remains insufficiently established, and microbiota-targeted interventions require further clinical confirmation.

## Figures and Tables

**Table 2:** Comparative immuno-metabolic features of the gut–skin axis in AD and psoriasis.

Feature	Atopic Dermatitis	Psoriasis
Dominant immune axis	Th2/IL-4/IL-13 [151,152]	IL-23/Th17/IL-17 [208,209]
Gut dysbiosis pattern	↓ α-diversity (especially in pediatric population); change in β-diversity; ↑ *Enterobacterales*; ↓ SCFA producers [249,250,251,254]	change in β-diversity; inconsistent F/B ratio; dysbiosis highly dependent on variables such as age, comorbidity, diet [238,247,267]
Primary pathogenic driver	Genetic predisposition; early-life immune imprinting; environmental factors [153]	Genetic predisposition; environmental factors [210,211]
Cutaneous impact	Barrier failure [164,165]	Hyperproliferation; thickening and differentiation disorder of epidermis; [218]
Commonly described metabolite-mediated signaling	SCFA via GPCR binding and HDAC inhibition; tryptophan metabolites via AhR binding [206,207,273]	SCFA via GPCR binding and HDAC inhibition; tryptophan metabolites via AhR binding [78,203,233]
Key epithelial triggers	Alarmins (TSLP, IL-33, IL-25) [153,166,167,168,169]	DAMPs; LL-37 + DNA/RNA; IFN-α [208,216]
Key keratinocyte outputs	CCL17, CCL22, CCL26 [184,185,187,188]	CCL20, AMP, IL-36 [218,221,222,223]

**Table 3:** Selected clinical trials evaluating gut microbiota modulation as adjunctive therapy in chronic inflammatory skin disease.

Disease	Intervention Type	Specific Agent	Study Population	Key Clinical Outcome	References
Atopic dermatitis	Probiotic	*Lactobacillus rhamnosus*	Children with AD	Reduction in SCORAD score.	[320]
Atopic dermatitis	Probiotic	*Lactobacillus plantarum*	Children with AD	Improvement in SCORAD and inflammatory markers.	[321]
Atopic dermatitis	Probiotic	*Bifidobacterium lactis*, *Bifidobacterium longum*, *Lactobacillus casei*	Children with AD	Reduction in SCORAD and decreased use of topical steroids.	[322]
Atopic dermatitis	Probiotic	*Bifidobacterium lactis*, *Bifidobacterium longum*, *Lactobacillus casei*	Children with AD	Reduction in SCORAD and decreased use of topical steroids.	[323]
Atopic dermatitis	Probiotic	*Lactobacillus plantarum*	Adults with AD	Reduction in SCORAD.	[324]
Atopic dermatitis	Probiotic	*Lactobacillus rhamnosus*, *Lactobacillus acidophilus*, *Lactobacillus paracasei*, *Bifidobacterium lactis*	Children and adolescents with AD	Reduction in SCORAD and reduced use of topical immunosuppressants.	[325]
Atopic dermatitis	Probiotic	*Lactobacillus plantarum*, *Lactobacillus reuteri*, *Lactobacillus rhamnosus*	Adults with AD	Reduction in SCORAD and inflammatory markers.	[326]
Psoriasis	Probiotic	*Bifidobacteria infantis*	Adults with psoriasis	Reduction in CRP and TNF-α levels.	[327]
Psoriasis	Probiotic + prebiotic	*Bacillus* spp. mix + FOS	Adults with psoriasis	Reduction in PASI and DLQI score.	[293]
Atopic dermatitis	FMT	Donor-derived fecal microbiota	Adults with AD	Improvement in EASI score and EASI-50 response.	[304]
Atopic dermatitis	FMT	Donor-derived fecal microbiota	Adults with AD	Reduction in SCORAD and decreased use of topical steroids.	[328]
Atopic dermatitis	Postbiotic	Oral bacterial lysate (*Escherichia coli*, *Enterococcus faecalis*)	Infants at risk of atopy	Reduced incidence of AD in subgroup with atopic predisposition.	[329]

Abbreviations: AD, Atopic Dermatitis; CRP, C-Reactive Protein; DLQI, Dermatology Life Quality Index; EASI, Eczema Area and Severity Index; FMT, Fecal Microbiota Transplantation; FOS, Fructooligosaccharide; PASI, Psoriasis Area and Severity Index; SCORAD, SCORing Atopic Dermatitis; TNF-α, Tumor Necrosis Factor-α.

## Data Availability

No new data were created or analyzed in this study.
